# Corrosion and Microstructural Investigation on Additively Manufactured 316L Stainless Steel: Experimental and Statistical Approach

**DOI:** 10.3390/ma15041605

**Published:** 2022-02-21

**Authors:** Héctor Maicas-Esteve, Iman Taji, Marc Wilms, Yaiza Gonzalez-Garcia, Roy Johnsen

**Affiliations:** 1 Department of Mechanical and Industrial Engineering (MTP), Faculty of Engineering (IV), Norwegian University of Science and Technology (NTNU), Richard Birkelandsvei 2B, 7034 Trondheim, Norway; iman.taji@ntnu.no (I.T.); roy.johnsen@ntnu.no (R.J.); 2 Shell Technology Centre Amsterdam (STCA), Grasweg 31, 1031 HW Amsterdam, The Netherlands; marc.wilms@shell.com; 3 Materials Science and Engineering Department, 3mE Faculty, Technical University of Delft, Mekelweg 2, 2628 CD Delft, The Netherlands; y.gonzalezgarcia@tudelft.nl

**Keywords:** selective laser melting, additive manufacturing, 316L SS, pitting corrosion, processing parameters

## Abstract

The use of metal additive manufacturing (AM) has strongly increased in the industry during the last years. More specifically, selective laser melting (SLM) is one of the most used techniques due to its numerous advantages compared to conventional processing methods. The purpose of this study is to investigate the effects of process parameters on the microstructural and corrosion properties of the additively manufactured AISI 316L stainless steel. Porosity, surface roughness, hardness, and grain size were studied for specimens produced with energy densities ranging from 51.17 to 173.91 J/mm^3^ that resulted from different combinations of processing parameters. Using experimental results and applying the Taguchi model, 99.38 J/mm^3^ was determined as the optimal energy density needed to produce samples with almost no porosity. The following analysis of variance ANOVA confirmed the scanning speed as the most influential factor in reducing the porosity percentage, which had a 74.9% contribution, followed by the position along the building direction with 22.8%, and finally, the laser energy with 2.3%. The influence on corrosion resistance was obtained by performing cyclic potentiodynamic polarization tests (CPP) in a 3.5 wt % NaCl solution at room temperature for different energy densities and positions (*Z* axis). The corrosion properties of the AM samples were studied and compared to those obtained from the traditionally manufactured samples. The corrosion resistance of the samples worsened with the increase in the percentage of porosity. The process parameters have consequently been optimized and the database has been extended to improve the quality of the AM-produced parts in which microstructural heterogeneities were observed along the building direction.

## 1. Introduction

Additive Manufacturing (AM) provides the capability to relax the design and manufacturing constraints by creating products with great geometrical complexity and without the need for extensive machining. After almost four decades of research and development, AM has proved to be a remarkable manufacturing technique, with an ongoing optimization of design methodology, material variety, and processing. Today, metal components fabricated with AM have obtained the mechanical properties and corrosion resistance required by aerospace, automotive, biomedical, energy, and marine applications [1,2]. Furthermore, researchers have demonstrated that the AM technology generates significant cost savings compared to conventional manufacturing due to the reduction of lead times and less waste of raw materials. Reduction in storage capacity for spare parts is another main advantage [3].

The AM technique allows for the creation of three-dimensional shape structures through a layer-by-layer process [4]. Three-dimensional computer-aided design (CAD) programs are used to design components with complex shapes that could not be manufactured via conventional processes such as casting or forging [5]. Among the AM techniques, selective laser melting (SLM) is the most versatile, allowing for the creation of functional parts with mechanical properties similar to those of conventionally produced materials [6]. The wide variety of materials that can be produced, together with the low surface roughness achieved, are what differentiates this technique from other production methods [7]. The building process is achieved by the successive consolidation of molten powder layers. A high-temperature laser beam melts the powder of the first layer (from 0.02 mm to 0.1 mm) [4]. The second layer of metal powder is spread out over the surface, and the same operation is repeated until the building process is completed. However, this technique still presents some challenges that must be addressed in order to improve the performance of the manufactured parts [8]. The main process parameters are the scanning speed ($v_{s})$; laser power ($P_{eff}$); hatch spacing (h); and layer thickness (d′). A correct combination of those parameters is crucial to achieving good quality for the final part. The combined action of these parameters can be reduced to the energy density ($E_{d}$) in J/mm^3^, which is the input energy received by the sample during the AM process, represented in Equation (1).

(1) $$E_{d}=\frac{P_{eff}}{v_{s}\times h\times d\prime }$$

Microstructural features, such as cellular structure, surface roughness, porosity, and residual stresses, are known to be affected by these processing parameters. In the work of J. Liu et al. [9], smaller and more elongated cellular structures were found when increasing the scanning speed from 800 to 1000 mm/s. Shang et al. [10] also evidenced grain refinement when increasing scanning speeds from 900 to 1083 mm/s. Relatively high surface roughness values of ~16 µm were observed for energy densities lower than 70 J/mm^3^, which was attributed to a lack of melting [11]. That surface roughness can be improved by re-scanning the surface, which also improves the density of the part [12]. Similarly, porosity can be reduced by avoiding exceedingly low or high laser energy densities. Values around $E_{d}\;$ = 100 J/mm^3^ have been reported in previous works [11,13] as optimal for reducing porosity levels. High energy densities promote gas formation and solidification shrinkage, increasing the level of porosity as well [11,14]. Residual stresses have also been the object of study for other researchers. Y. Liu et al. [15] proved that higher residual stresses correlated with high energy inputs. The fact was attributed to the shrinkage of the molten pool after being solidified. D. Wang et al. [16] focused on how the different scanning strategies affect the residual stress levels, concluding that a spiral divisional strategy reduces residual stresses and the deformation of the parts.

In the same way, the corrosion resistance of the parts produced by using powder bed fusion techniques (PBF) has been a topic of interest in numerous studies [17,18,19,20]. In the austenitic 316 stainless steels, corrosion started when the passive layer rich in chromium oxide Cr_2_O_3_ degraded locally, which resulted in the creation of small pits. The potential associated with this process is known as the pitting potential (*E_pit_*) or breakdown potential (*E_break_*). Pores, inclusions, grain boundaries, and scratches in the oxide layer are known as precursors of the protective film loss [21]. Determining how the process parameters influence the initiation factors that promote this form of localized corrosion is still not well-defined. Suryawanshi et al. [19] stated that the presence of pores on the surface causes the preferential migration of chloride ions. A diffusion barrier is created around the top of the pores, which leads to a higher concentration of aggressive agents, thus causing a reduction in the corrosion resistance of AM samples as compared to the wrought ones [18]. The work of Sander et al. [5] revealed a strong correlation between the process parameters and the specimen porosity responsible for the decreased repassivation ability. A better understanding of the effect of the process parameters is important in applications that require parts that are highly resistant to corrosion, such as those in the aerospace industry, opening up the possibility of replacing costly production methods such as diffusion bonding or TLP bonding, which are used for the production of turbine engines [22].

The AM production methods have been reported to provide superior properties as compared to other production techniques. For example, AM specimens have higher density levels, lower frictional wear, and higher hardness than specimens created by powder metallurgy [23], and higher corrosion resistance than samples created by spark plasma sintering due to their reduced porosity level [24]. However, in the production of AM components, several existing techniques do not produce samples with the same mechanical and microstructural properties due to the use of different instrumentation. In fact, the parts created using the same production technique exhibit microstructural properties that vary significantly from each other, since there are a few parameters that affect the production process and consequently the final quality of the product. In the previous literature, many researchers have been modifying these input parameters, trying to find the optimal combination in order to improve the microstructural properties of the parts. Gor et al. [25] provided a review of the state-of-the-art the effects of process parameters in AM parts. It can be noted from this review that the range of applicable energy densities required for the improvement of certain material properties is very wide. Energy densities ranging from 50 to 150 J/mm^3^ are needed to improve the tensile strength, between 80 and 125 J/mm^3^ to increase the hardness of the samples, or from 80 to 105 J/mm^3^ to reduce porosity levels in the AM components. Sun et al. [18] used a laser powder of 150 W, a layer thickness of 0.05 mm, and different scanning speeds ranging from 125 to 200 mm/s to study the effect on porosity. The scanning speed of 150 mm/s proved to be the optimum among those studied to reduce the amount of porosity. Lower and higher scanning speeds increased the percentage of porosity, with a significant increase in the number of pores for the sample produced at the higher scanning speed (200 mm/s). This fact was attributed to the reduced energy input received by the sample, resulting in reduced melting of the molten pool. Liverani et al. [26] produced the samples for the mechanical and microstructural characterization studies, applying laser powers of 100 and 150 W; hatch spacings of 0.05 mm and 0.07 mm; while keeping the scanning speed fixed at 700 mm/s. Their results showed that laser power had a strong influence on the amount of porosity of the samples. The combination of 150 W and 0.07 mm was found to be optimal for producing samples with high relative densities >99%. In contrast, the combination of the lower values of laser energy and hatch spacing (100 W and 0.05 mm) yielded the lowest density levels in the samples. On the other hand, Mugwagwa et al. [13] carried out studies of the residual stresses on samples produced via SLM, using scanning speeds of 200 and 1000 mm/s, and laser powers between 80 and 180 W with 20 W increments. The results showed that the highest relative density of 99.58 % was achieved with the combination of 600 mm/s and 180 W.

Some of the previous researchers have changed certain parameters while leaving others unchanged, and vice versa, testing specific ranges and hence leaving a large amount of the values without studying their effect. Hanzl et al. [27] studied the influence of the scanning speed on the material structure, varying its value from 50 mm/s to 80 mm/s for a fixed laser power of 300 W. A denser and more consistent structure was observed when using 50 mm/s. However, a structure in which coarse spherical agglomerates were predominant was found when the scanning speed was increased to 80 mm/s. Shang et al. [10] focused the production of the samples on three scanning speeds—800, 900, and 1083 mm/s; laser powder at 195 W; hatch spacing at 0.09 mm; and a layer thickness of 0.02 mm. The experiments showed a remarkable increase in porosity for the samples produced with 1083 mm/s due to the insufficient fusion of the powder particles, which led to a decreased corrosion resistance. Li et al. [28] observed that with the increase of the scanning speed from 90 mm/s to 180 mm/s, the molten pool size and the density gradually decreased. In those experiments, the laser power was kept constant at 100 W and the layer thickness at 0.06 mm. All these combinations of process parameters were chosen at the discretion of each of the authors of these experiments. Therefore, a larger database is necessary to be able to decide how to produce these materials for future industrial applications. The approach of this database should be in the way of combining several different production parameters without limitations in order to be more general and logical. These combinations will be reflected in the energy density received by the sample. 

This article proposes the use of a statistical approach to find the most effective parameter in the production method and to weight other parameters for corrosion and mechanical properties as well. In this way, the producer will know which parameter is more crucial to play with if they need a specific property. The statistics approach presented in this article is also verified with results from experimental tests.

This study aims to establish the corrosion properties of the AISI 316L stainless steel (316L SS) produced via SLM as compared to conventional production techniques; to explain the corrosion properties of produced components in terms of microstructure and parameters involved in the production process, such as laser energy and scanning speed; and to provide recommendations for process parameters in order to optimize the corrosion resistance of AM processes. A statistical design of experiments (DoE) has been used to study the relationships between parameters and their responses. DoE is defined as a systematic method that describes the relationship between the factors or inputs and the output or responses of a process [29]. When working with numerous input factors, they are widely used to effectively control the interactions between them while optimizing the number of experimental runs. Concretely, the Taguchi method was chosen as the fractional factorial design for conducting the experiments. This method, based on orthogonal arrays, considers the effect of the different factors independently and at the same level prior to starting the experimental runs [30]. In those balanced arrays, factors and levels are combined to assess the weight/effect on a given output.

## 2. Materials and Methods

### 2.1. Materials

**Figure 1:**
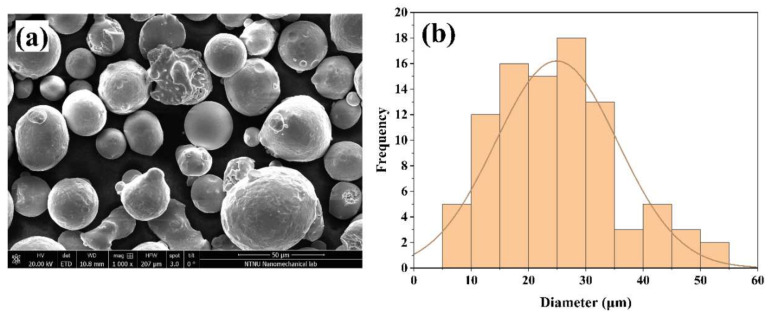
SEM image of GE powder at 1000× (**a**) and histogram showing the powder size distribution (**b**).

Two manufacturing processes were chosen to produce the specimens: additively manufactured (AM) with SLM and conventionally manufactured (CM) wrought 316L SS. The AM stainless-steel specimens were produced in Amsterdam (The Netherlands) at Shell Technology Centre Amsterdam (STCA) in an M2 dual laser machine under argon atmosphere, using 316L metallic powder from General Electric (GE), with a particle size distribution (PSD) of 15–45 μm provided by the supplier. Powder particles were examined under the scanning electron microscope (SEM) produced by FEI (Hillsboro, OR, USA), and its characteristic percentile values were D_10_: 12 μm, D_50_: 24 μm, and D_90_: 41 μm. These morphological data indicate the powder sizes whose amounts were found to be below 10%, 50% and 90% of the total number of particles (Figure 1). Specific details of the compositional elements of the AM 316L SS are presented in Table 1. For the commonly manufactured samples (wrought), a 40 mm diameter round bar from the company Roldan, S.A. - Stainless Steels located in León (Spain) was used. Both, AM and CM samples subjected to the microstructural and corrosion studies were previously metallographically prepared by surface grinding to a #2000 grit surface finish using silicon carbide paper and polished up to 3 μm with diamond paste. Before testing, they were immersed in an ultrasonic bath for 5 min and then cleaned with ethanol to remove possible remaining particles on the surface. Only samples submitted to an electron backscatter diffraction analysis (EBSD) performed in the SEM, produced by FEI (Hillsboro, OR, USA), were electropolished at 30 volts for 30 s in a mixture of sulfuric acid and methanol (5.3–94.7 vol %).

The AM 316L SS specimens were built with different energy densities resulting from the combination of three laser powers, 150/180/200 W, at three scanning speeds, 400/700/1000 mm/s. The optimal energy density proposed by the powder supplier was taken as a reference when choosing the levels of the different parameters for this study. This corresponded to 89.44 J/mm^3^, resulting from the combination of a laser energy of 180 W with a scanning speed of 700 mm/s. The objective was to have a wide range of energy densities to study, both above and below the suggested energy density by the supplier. A total of nine experimental runs were carried out in this study, and for each of them, a different combination of process parameters was used, as shown in Table 2. The parameters set as fixed were the spot diameter (0.140 mm), the hatch spacing (0.115 mm), and the layer thickness (0.025 mm). The scanning strategy was also fixed during the process, characterized by a continuous exposure pattern of bidirectional and alternating scanning vectors 0°45°. No post-heat treatment was applied.

**Figure 2:**
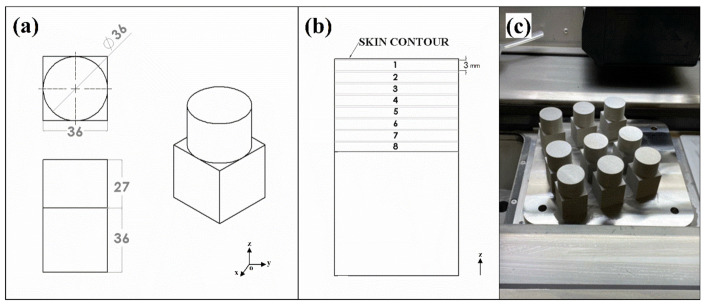
Three-dimensional sketch of the AM specimens in mm (**a**,**b**); AM specimens on the building platform after being produced (**c**).

The shape of the AM specimens consisted of cylinders (36 × 27 mm) mounted on top of cubes (36 × 36 mm) rising from the building platform in the direction of the *Z* axis, as shown in Figure 2a. Different positions along the building direction were selected from the previously divided specimens to investigate the effect of the building position on the microstructural/corrosion properties (Figure 2b). The AM samples were identified with the use of two numbers (e.g., 3.2), the first one corresponding to the applied energy density and the second to the position along the *Z* axis.

The nine AM 316L SS specimens (Figure 2c) were cut into 3 mm discs for the subsequent microstructural and corrosion investigation with Electro Discharge Machining (EDM) produced by ONA (Durango, Spain). To perform surface roughness analysis, samples from the top of the cylinders were cut with the outer skin (e.g., 3 mm + skin contour thickness).

### 2.2. Microstructural Studies

#### 2.2.1. Porosity/Pore Measurement

Five images were taken for each sample at different locations with the Hirox RH 2000 digital microscope produced by Hirox Co. Ltd. (Tokyo, Japan). For each image, the percentage of porosity was analyzed using the ImageJ software. The average porosity level of the five images determined the porosity percentage for that position. The pore size was also analyzed similarly as for the porosity study.

#### 2.2.2. Roughness (Ra)

Only the top cylindrical samples were examined under the microscope for the roughness analysis. For each sample, three Ra measurements were taken along a 10 mm length in the center of the samples using the “3D profile” tool of the Hirox RH2000 digital microscope. The cutoff values were adjusted, eliminating the remaining traces of waviness. The results were plotted and processed using the Origin lab software.

#### 2.2.3. Hardness

The hardness for all the energy densities was measured for the central position (#4). The study was carried out using the Mitutoyo hardness testing machine produced by Mitutoyo (Sakado, Japan). Five micro-indentations were made in each sample at different locations to obtain the average hardness value (HV). The applied strength was 1 Kgf in a sequence of 4/15/4 s (approaching /loading/unloading).

#### 2.2.4. Grain Size

The grain sizes were studied for the central positions (#4) of the energy densities 52.17, 89.44, and 173.91 J/mm^3^. The samples were prepared by electropolishing to obtain the smooth surface required by electron backscatter diffraction (EBSD) analysis performed in the SEM (FEI Quanta FEG 650, Environmental SEM) produced by FEI (Hillsboro, OR, USA). The electropolishing was applied at 30 volts for 30 sec in a mixture of sulfuric acid and methanol (5.3–94.7 vol %). Information about crystallographic orientations and strain level due to grain deformation were obtained through inverse pole figures (IPF) and kernel average misorientation analysis (KAM), respectively.

### 2.3. Corrosion Evaluation

Samples corresponding to the positions #2, #5, #8 (top-middle-bottom) and energy densities 52.17, 89.44, 173.91 J/mm^3^ were selected for corrosion testing. More specifically, cyclic potentiodynamic polarization tests (CPP) were carried out twice to ensure the reproducibility and consistency of the results.

The CPP measurements were conducted in the AVESTA electrochemical cell (BioLogic). During the test, a surface area of 1.5 cm^2^ was exposed to an electrolyte of a sodium chloride (NaCl) solution (3.56 wt %; 0.6 M) in distilled water. A silver–silver chloride electrode (Ag/AgCl) was used as a reference electrode, and a platinum mesh was used as a counter electrode. All potentials given later refer to this reference electrode. 

The electrolyte for the test was purged with nitrogen gas for one hour in a separatory funnel. At the same time, nitrogen gas was introduced into the AVESTA cell, creating an oxygen-free atmosphere. After an hour, 160 mL of electrolyte was transferred to the AVESTA cell, where the electrolyte was continuously purged with nitrogen until the test was completed. The oxygen values during the test at room temperature never exceeded 10 ppb. Each sample was stabilized in the electrolyte for 1 h while measuring the Open Circuit Potential (OCP). Cyclic potentiodynamic polarization was then performed at a scanning rate of 0.1667 mv/s, starting from −0.15 V vs. OCP according to the ASTM G61. The direction of the scan was reversed at 0.1 mA/cm^2^. The voltage during the test was applied and controlled by the Interface1000 Gamry potentiostat, and the polarization curves were plotted in Excel. Energy-dispersive X-ray spectroscopy analysis (EDS) was performed inside the pits to determine the chemical composition at different spots of the attacked area.

### 2.4. Statistical Analysis

A statistical design of experiments (DoE) was carried out to study the relationships between parameters and their responses. More specifically, the Taguchi method was chosen as the fractional factorial design for conducting the experiments. Taguchi L9 orthogonal array (OA), with 3 factors and 3 levels for each individual factor, defined the sequence of experiments for the porosity study (Table 3).

## 3. Results

### 3.1. Roughness Measurements

**Figure 3:**
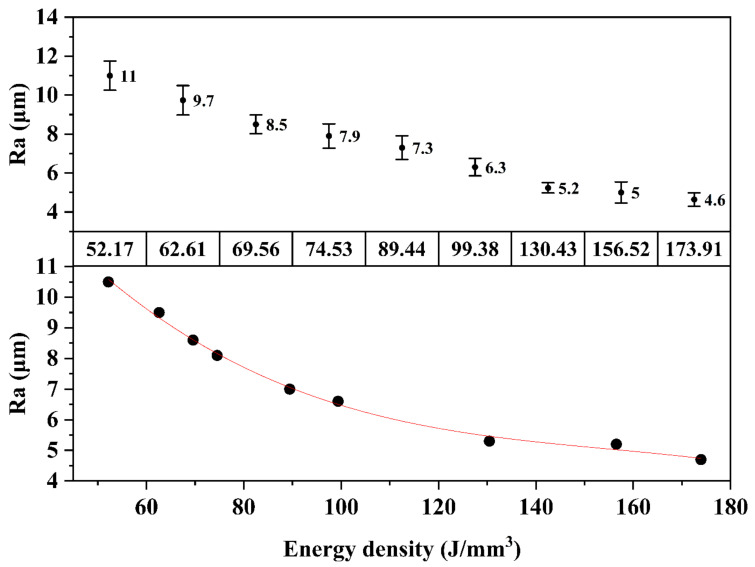
Evolution of the average roughness (Ra) of the top samples and the fitted model when increasing energy densities.

**Figure 4:**
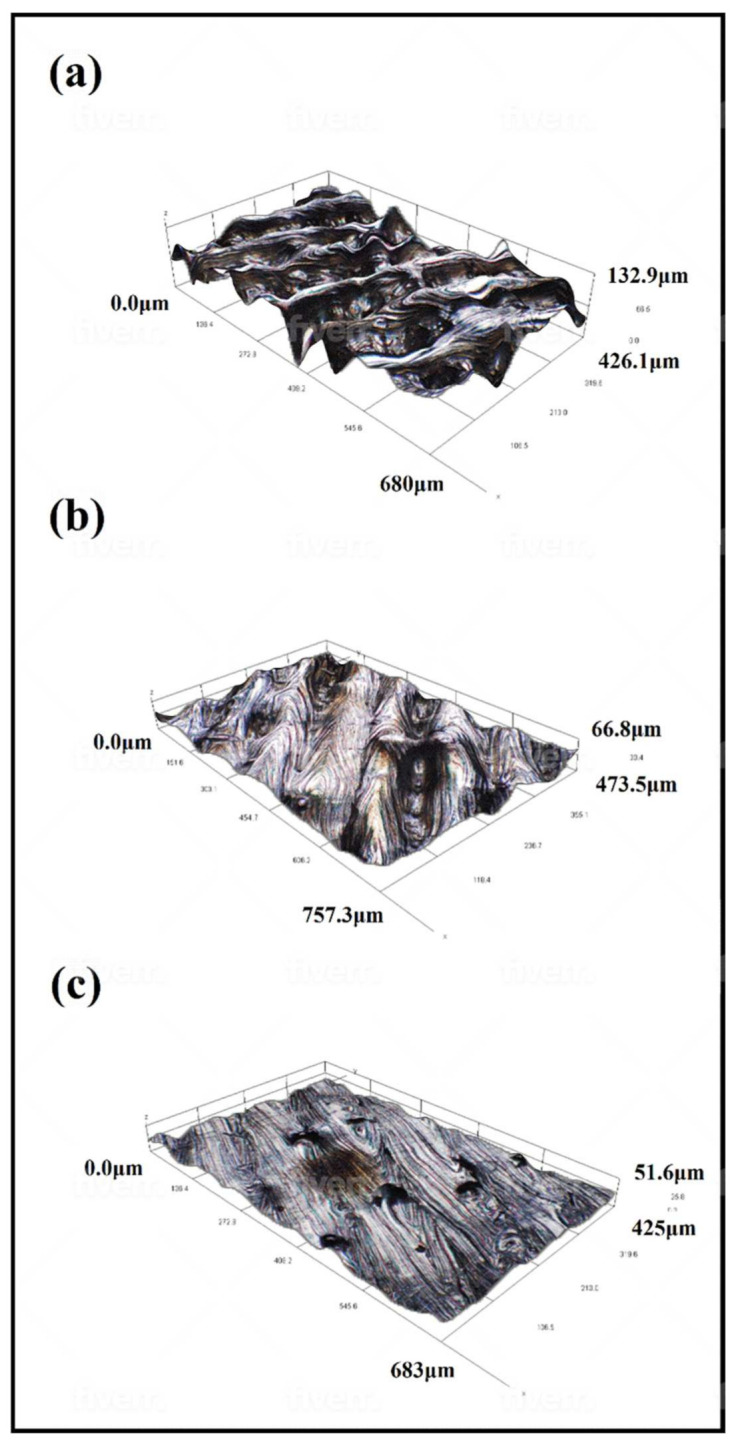
Three-dimensional surface roughness profile for densities of 52.17 J/mm^3^ (**a**); 89.44 J/mm^3^ (**b**); and 173.91 J/mm^3^ (**c**).

The roughness study showed a decrease in Ra with increasing energy density. This happened progressively for all energy densities, reaching a minimum Ra value of 4.6 for the energy density of 173.91 J/mm^3^, as shown in Figure 3. A regression analysis of the results was then conducted to describe its evolution. The R^2^ of the adjusted model was 99.7%, so it adequately represented the relationship between roughness and energy density used during the process. Figure 4 shows a detail of the top surface profile, evidencing for each energy density the different depths of the scanning tracks.

The regression Equation (2) of Ra from the fitted model was:

(2) $$Ra\;\left(\mu m\right)=21.134-0.282\;E_{d}+0.00173\;E_{d}{}^{2}-3.714e^{-6}\;E_{d}{}^{3}\;\left(R^{2}=99.7\%\right)$$

### 3.2. Hardness Measurements

**Figure 5:**
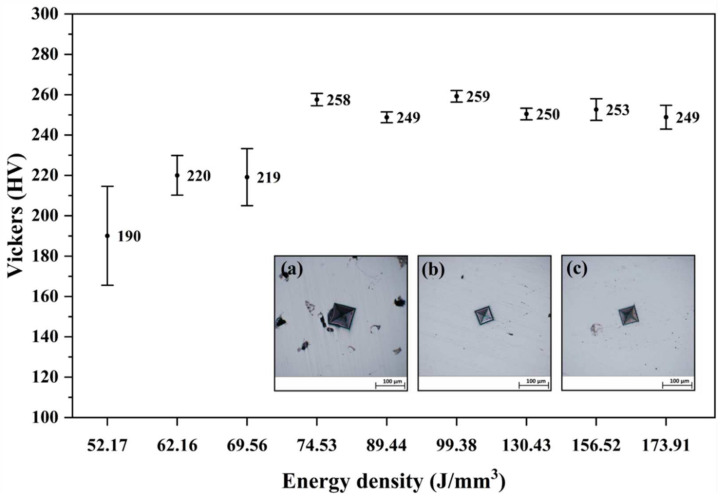
Evolution of hardness (HV) for the position (#4) when increasing energy densities. Micro-indentation void in the AM samples produced with densities of 52.17 J/mm^3^ (**a**); 89.44 J/mm^3^ (**b**); and 173.91 J/mm^3^ (**c**).

The minimum average hardness value of 190 HV was found for the samples built with the lowest energy density of 52.17 J/mm^3^. From this point on, the hardness increased progressively up to the energy density of 74.53 J/mm^3^. These energy densities exhibited high dispersion at the values obtained from the different indentations, which were clearly influenced by the high porosity content, as shown in Section 3.3. Higher porosity was found in the samples at lower energy densities due to the incomplete melting of the powder. Therefore, the probability of indenting into a pore increased. For higher energy densities, the hardness remained stable at around an average value of ~253 HV, while reaching its maximum of 259 HV for the energy density of 99.38 J/mm^3^, as shown in Figure 5.

### 3.3. The Effect of Energy Density and Building Positions on Porosity

**Figure 6:**
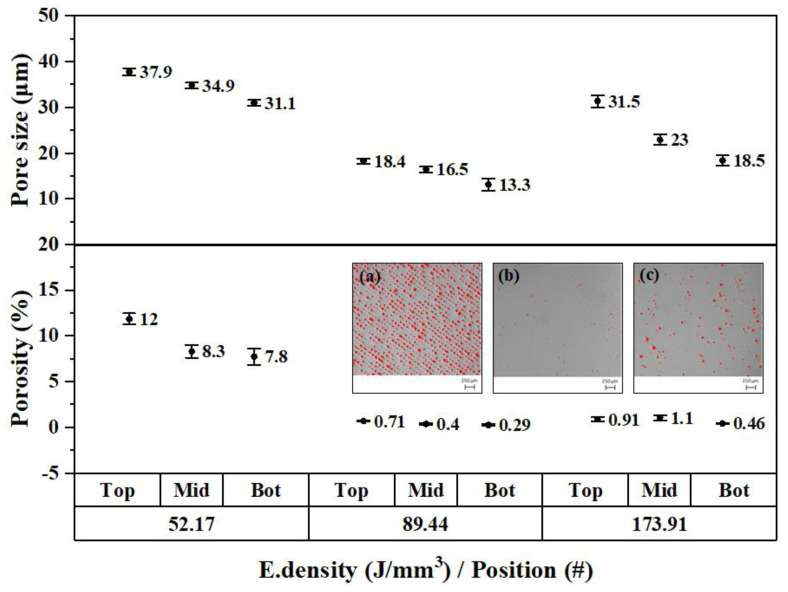
Pores sizes and porosity (%) for the energy densities 52.17, 89.44, and 173.91 J/mm^3^ and positions 2, 5, and 8. Digital microscope images of the surfaces showing the porosity content of 52.17 J/mm^3^ (**a**); 89.44 J/mm^3^ (**b**); and 173.91 J/mm^3^ (**c**).

The porosity study revealed that the position of the sample relative to the *Z* axis has an influence on the obtained porosity. Samples from top positions exhibited higher porosity than samples closer to the building platform. The mean pore size also followed the same trend as that of porosity, decreasing for positions closer to the printing bed, as shown in Figure 6. The largest percentage of porosity and pore size was found for the energy density of 52.17 J/mm^3^, followed by 173.91 J/mm^3^, and finally, for the optimal energy density suggested by the powder supplier, 89.44 J/mm^3^. 

**Figure 7:**
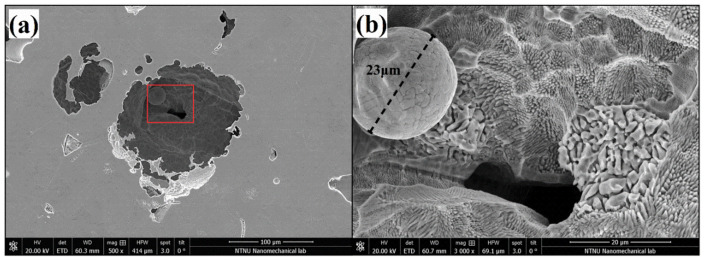
SEM image of a pit at 500× (**a**) and 3000× magnification (**b**), showing the lack of fusion in the AM sample 3.5 (52.17 J/mm^3^; middle position).

Unmelted particles were also discovered within the pores for the density of 52.17 J/mm^3^, as shown in Figure 7. This microstructural defect occurred due to the incomplete melting of the powder when exceedingly low energy densities were used [20].

### 3.4. Grain Size and Internal Stress

**Figure 8:**
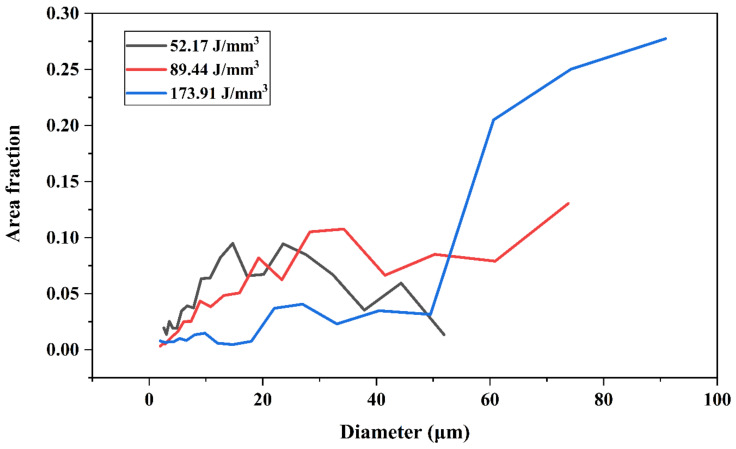
Area fraction of grain size diameters for energy densities of 52.17, 89.44, and 173.91 J/mm^3^.

From the Electron Backscatter Diffraction analysis (EBSD), information about grain size and misorientation angle for different samples was obtained. The grain size distribution for the energy density of 52.17 J/mm^3^ followed a normal distribution curve, with two remarkable peaks at 15 μm and 24 μm, where the maximum area fraction of ∼0.09 was observed, as shown in Figure 8. On the other hand, for the optimal energy density of 89.44 J/mm^3^, the curve followed a more linear trend, reaching a maximum area fraction of ~0.13 for grain sizes of around 74 μm. Grain sizes of 28 μm and 34 μm should be highlighted in the same way at this energy density since they also reached significant area fractions of ~0.10. For the highest energy density of 173.91 J/mm^3^, the evolution of grain sizes was exponential. Up to 50 μm of the area fraction remained very low. However, from this value onwards, it increased drastically, reaching a maximum of 0.277 for grain sizes of around 91 μm. 

**Figure 9:**
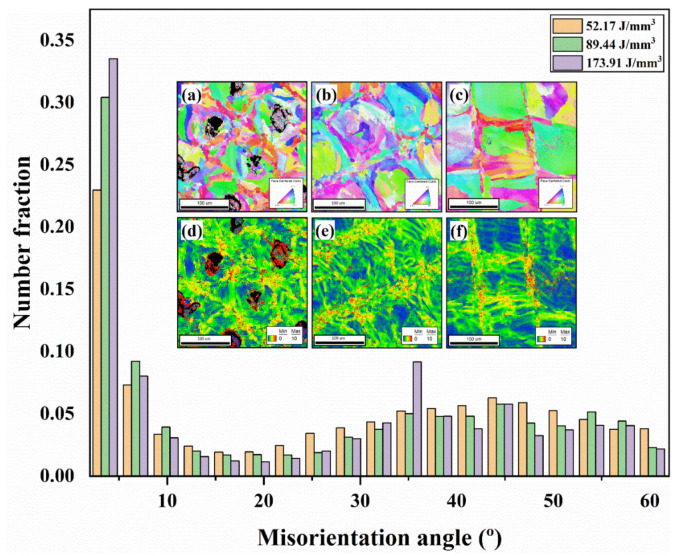
Number fraction of misorientation angles, with IPF (**a**–**c**) and KAM (**d**,**c**,**f**) figures from the EBSD analysis, for the energy densities of 52.17 J/mm^3^ (**a**,**d**); 89.44 J/mm^3^ (**b**,**e**); and 173.91 J/mm^3^ (**c**,**f**).

A remarkably high fraction of low-angle grain boundaries (LAGBs) was found in all samples, as seen in Figure 9. This is commonly found in AM samples [31]. However, this fraction increased as the energy density increased. The Kernel Average Misorientation (KAM) of the corresponding EBSD images are depicted in Figure 9. The KAM analysis represents the lattice misorientation of every single point relative to the fifth neighboring points. The higher the internal stress, the more distortion would be expected in the lattice. The distortion degree is represented by the colors in Figure 9d–f. The blue color represents the base lattices without distortion, while the degree of lattice distortion is represented by the green, yellow, and red colors (where red indicates the highest distorsion and green the lowest). As it can be seen, the lattice distortion and hence the internal stress increased by decreasing the energy density.

### 3.5. Corrosion Results

#### 3.5.1. Effect of Building Position on Corrosion

**Figure 10:**
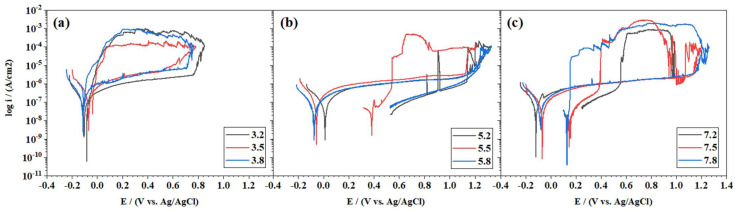
CPP curves for AM samples with different positions 2, 5, and 8, and energy densities of 52.17 J/mm^3^ (**a**); 89.44 J/mm^3^ (**b**); and 173.91 J/mm^3^ (**c**) in a 3.56 wt % NaCl solution at room temperature.

The corrosion behavior of samples with different positions has been studied to elucidate the effect of position on the corrosion resistance. It has been demonstrated in previous sections that the position along the *Z* axis had a strong impact on the microstructure of the samples. An increased percentage of porosity was found for samples located at higher positions in the building direction. Therefore, the positions #2, #5, and #8 were studied for the energy densities of 52.17, 89.44, and 173.91 J/mm^3^. The CPPs corresponding to the different positions are represented in Figure 10.

Sample 3.2 shows the most positive breakdown potential (E = 0.76 V) of the group produced with an energy density of 52.17 J/mm^3^ and the lowest passive current density (i_pass_ = 1.63 × 10^−6^ A/cm^2^). This sample exhibited the highest percentage of porosity at 12% and a grain size of 37.9 μm among those of its group. Samples 3.5 and 3.8 showed higher i_pass_ and lower breakdown potentials than sample 3.2. The repassivation capacity for this group of samples was very low. The repassivation potentials, E_rep_, intercepted the forward scanning outside the passive region close to the cathodic branch. In the second group (89.44 J/mm^3^), the samples were characterized by a transpassive breakdown at high electrochemical potentials (~1.1 V). Samples 5.2 and 5.8 were the most corrosion-resistant, showing almost no pitting corrosion. Sample 5.5 evidenced higher pitting damage. In that sample, the passive layer did not recover until it reached a potential close to E_rep_ = 0.54 V. All samples produced with the highest energy density of 173.91 J/mm^3^ exhibited pitting corrosion. However, they showed high E_pit_ values (~1 V). The total recovery of the passive layer occurred in a staggering order of positions: top position 7.2 (E_rep_ = 0.55 V), middle position 7.5 (E_rep_ = 0.39 V), and bottom position 7.8 (E_rep_ = 0.15 V). In this group, the repassivation ability contradicted the porosity results as it was better for the top sample when there was more porosity and decreased as the porosity was reduced. However, the porosity percentage difference in this group is small and does not alter too much between samples.

The effect of the positions within each group did not greatly impact corrosion resistance. A repetitive trend among different groups of energy densities could not be observed either. Table 4 shows the characteristic corrosion parameters for the different groups, where E_corr_ was obtained by the Tafel extrapolation method.

#### 3.5.2. Effect of Energy Density on Corrosion

**Figure 11:**
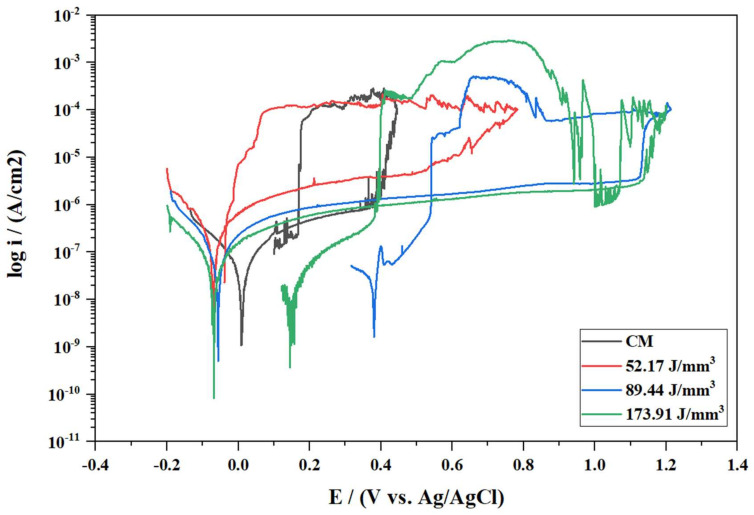
CPP curves for CM and AM samples with energy densities of 52.17, 89.44, and 173.91 (J/mm^3^) and position (#5) in a 3.56 wt % NaCl solution at room temperature.

The CPPs for the central position samples (#5) belonging to three different energy densities: 52.17, 88.44, and 173.91 J/mm^3^, are plotted in Figure 11. In this figure, the corrosion behavior of the AM samples and the CM sample was compared.

Sample 3.5, built with the lowest energy density of 52.17 J/mm^3^, exhibited the lowest corrosion resistance among the AM samples. It was characterized by having a higher passive current density (i_pass_ = 3.91 × 10^−6^ A/cm^2^) and a lower breakdown potential (E_pit_ = 0.51 V) than the rest of the samples.

On the other hand, sample 5.5, produced with the energy density of 88.44 J/mm^3^ suggested by the powder supplier, showed much higher corrosion resistance. The potential in the anodic branch tended to the transpassive region (E_pit_ = 1.13 V), although damage caused by pitting corrosion was evident under microscope examination. The sample presented higher repassivation ability than the others by reaching the repassivation potential earlier (E_rep_ = 0.54 V) in the hysteresis loop. 

Finally, sample 7.5, built with the highest energy density of 173.91 J/mm^3^, exhibited similar corrosion characteristics to that produced with a density of 88.44 J/mm^3^. When the breakdown potential was reached, pitting damage was evident; however, the passive layer eventually recovered, as shown in Figure 11. After this point, the pitting damage exceeded the repassivation ability, and pitting corrosion took place all over the surface. The passive layer was fully recovered at a repassivation potential of E_rep_ = 0.39 V. 

The studied CM wrought samples showed a lower corrosion resistance than those produced with SLM. A lower breakdown potential (E_pit_ = 0.37 V) as compared with the AM samples was observed and showed a higher level of metastable pitting.

### 3.6. Statistical Modelling

#### 3.6.1. Factor Interactions (Taguchi)

The porosity values corresponding to the combinations proposed by the statistical model are shown in Table 5, together with the signal-to-noise ratios (S/N) obtained from the Taguchi factor interactions. The S/N values are the ratios between the mean of the response and the standard deviation for each combination of factor levels involved in the design [32]. In order to obtain these ratios, a desired objective for the model must be established. In this case, it was desired to obtain the lowest porosity percentage in the samples. The corresponding formula for the “smaller-the-better” is represented in Equation (3).

(3) $$\mathrm{Smaller}-\mathrm{the}-\mathrm{better}=-10\;\log\frac{1}{n}\sum {\left(R\right)}^{2}$$

*R* = value of the response for a factor level

*n* = number of responses for a factor level

A higher S/N ratio represents the “minimum variation difference between the desired output and measured output” [33]. With the values represented in Table 6, it was possible to obtain the delta value, which is the highest mean value of the response minus the lowest mean value of the response for the different levels of that factor [34]. The delta value (Max–Min) was used to classify the level of influence of each parameter. Scanning speed was rated as the most influential parameter in the ranking, followed by position, and finally, the laser energy. The best combination to obtain the lowest porosity value, as revealed by the Taguchi model, was the one that combined 200 W, 700 mm/s, and position #5 (middle position).

#### 3.6.2. Confirmation Test for the Suggested Combination

The combination of parameters predicted by the Taguchi model as the best to reduce porosity levels in the samples was the one combining ($P_{eff}=200\;W;\;v_{s}=700\;mm/s\;;\;\#=5)$. This specific combination was not within the initial nine combinations selected in the Taguchi matrix for the porosity study. To verify the accuracy of the model, the suggested combination was subjected to the porosity study as well. The obtained porosity of 0.05% corroborated that the model was right in its prediction, suggesting the best combination of process parameters in order to obtain the lowest porosity percentage in the sample. With the suggested combination, a total reduction in porosity of 88.37% was achieved, as shown in Table 7, with respect to the combination suggested by the powder supplier.

#### 3.6.3. Analysis of Variance (ANOVA)

With the ANOVA, it was possible to calculate the percentage of contribution of each parameter to the porosity. The contribution was obtained by dividing the specific sum of squares of a factor over the total amount. As shown in Table 8, the biggest contribution was provided by the scanning speed with 74.86%, followed by the position with 22.83%, and finally, by the laser energy with 2.27%.

#### 3.6.4. Modelling (Regression Equation)

A mathematical model based on the Taguchi design was developed to predict the amount of porosity in the sample with respect to the energy density applied during the building process. The experimental data was fitted to a regression model using the regression analysis tool provided by Origin lab software. 

**Figure 12:**
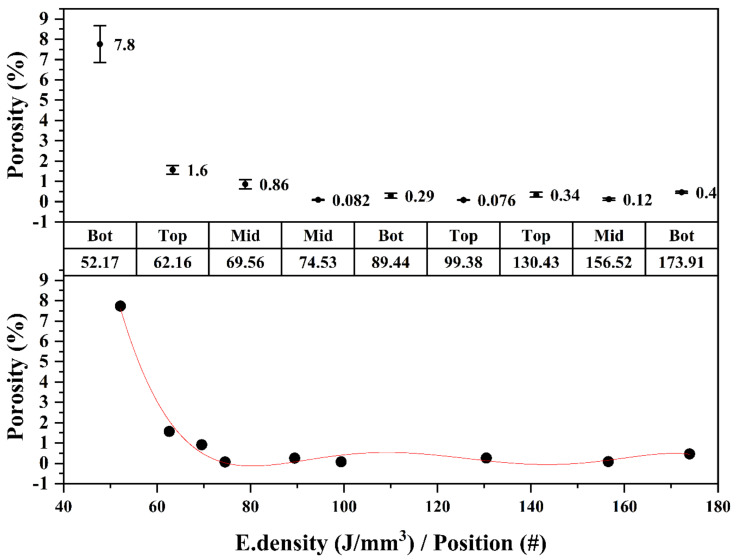
Average porosity (%) and fitted regression model when increasing energy densities.

As shown in Figure 12, the data was properly fitted with an $R^{2}$ of 98.74%. The regression equation that describes the vol % porosity vs. energy density is as follows:

(4) $$Porosity\;\left(\%\right)=216.73-9.6\;E_{d}+0.16536\;E_{d}{}^{2}-0.00138\;E_{d}{}^{3}+5.63e^{-6}\;E_{d}{}^{4}\;-8.956e^{-6}\;E_{d}{}^{5}\;\left(R^{2}=98.74\%\right)$$

To validate this model, some tested porosity values from the $L_{9}$ orthogonal matrix were randomly chosen, which are listed in Table 9. The values predicted by the model are quite close to the experimentally obtained results, although the model predicts some negative values of porosity when the content is too low.

Equation (4) accurately predicts the porosity values for different combinations of process parameters. This means that knowing the theoretical energy density that will be applied to the sample makes it possible to predict any production batch’s porosity.

## 4. Discussion

### 4.1. The Role of Building Position on the Microstructure and Corrosion Performance

The percentage of porosity decreased for positions closer to the printing platform. Top sample positions showed higher porosity percentages and pore sizes than samples at lower positions in the building direction (*Z* axis), as shown in Figure 6. This can be explained by the ununiform residual stress distribution present along the height direction, as discussed in the work of Y. Liu et al. [15]. When the laser advances over the surface, the molten pool begins to cool down. Once the temperature is lower than its plastic state, volume shrinkage in the melted area and tension stresses begin to be generated, which are constrained by the vicinity of the molten pool. In this area, the compression stresses increase to counteract the tension stresses that begin to form, which are transferred to areas far away from the heat-affected zone, deepening towards lower positions. When the part is at room temperature, tension stresses remain on top and compression stresses accumulate in lower positions, which may help to reduce the porosity and pore sizes. This agrees with what has been experienced in this work, providing a good explanation for the results obtained. The tension stresses located in the upper areas promote the thickening of the pore sizes; however, when going deeper to lower areas in the building direction, the accumulated compression stresses favor the creation of a more compact microstructure with less porosity and reduced pore sizes. This shows that residual stresses must be considered in the AM parts since they affect the microstructure to a significant extent.

On the other hand, the influence of position on corrosion resistance was not so evident. The corrosion resistance for the samples produced with the same parameters but with different positions on the *Z* axis did not vary too much. It should be noted that the greatest contrast in terms of microstructural properties was observed between groups of different energy densities. The microstructural variations between samples from the same group at different positions were remarkable but not enough to observe CPP curves with overly divergent corrosion behaviors. For low porosity levels of <1%, the E_pit_ values did not show any correlation with the specimen porosity, as previously reported by Sander et al. [17] for porosities of <0.5%. When this percentage exceeded 1%, a progressive reduction in corrosion resistance was observed. That trend was already seen in the works of Suryawanshi et al. [19] and Sun et al. [18] for porosities exceeding 1.4% and 1.7%, respectively. However, in previous studies [17,18,19], the corrosion tests were carried out on the cross sections of the samples (i.e., perpendicular to the building direction). This is a critical issue in the studies because they did not take into account possible variations in the porosity content along the *Z* axis. In this work, the corrosion tests were performed on sections parallel to the building direction, where sample porosities were shown to be influenced by the *Z* axis position Figure 10. For the studied positions (2, 5, 8) of the energy densities 52.17, 89.44, 173.91 J/mm^3^, the dispersion of the porosity values around the mean was confirmed by the results shown in Table 4. It was proven that even upon finding those deviations, there was similar resistance to corrosion throughout the structure. But perhaps for larger structures, these variations are more noticeable, and therefore the corrosion resistance can differ between different zones of the same component.

### 4.2. The Energy Density Influence on the Microstructure and Corrosion Performance

The highest percentages of porosity and pore size were found in the sample produced with the lowest energy density of 52.17 J/mm^3^. In the work of Cherry et al. [11], a peak porosity of 8.8% was obtained when using the lowest energy density of 41.81 J/mm^3^. By increasing the laser energy density to 104.54 J/mm^3^, the porosity decreased to 0.38%. When increasing the energy density, the viscosity decreases, improving the fluency of the melted powder and densifying the part. That value of porosity increases again due to the vaporization of the powder when its temperature is increased about twice the material melting temperature, as stated by Campanelli et al. [14]. Gases such as CO and CO_2_ are released due to the chemical reaction produced by high temperatures [10]. In this work, a rebound in the percentage of porosity was also observed when the applied energy density was increased from 89.44 to 173.91 J/mm^3^, as shown in Figure 6. In the work of Mugwagwa et al. [13], an energy density of 99.58 J/mm^3^, almost identical to the one suggested by the Taguchi model (99.38 J/mm^3^), was reported as the optimal to produced samples with a higher relative density of 99.58% (i.e., porosity < 0.5%). The energy density also had a big impact on the pore sizes, increasing the diameters for energy densities that were too low and too high. In the work of J. Liu et al. [9], more than 80% of the pores had a diameter in the range of 10–20 μm when applying energy densities between 80–111 J/mm^3^. Similar pore size diameters (13–19 μm) were obtained in this work for an energy density of 89.44 J/mm^3^. The remarkable fact revealed in this study is that finding the optimal energy density does not ensure a uniform porosity content evenly distributed throughout the structure. Previous studies did not consider this [11,13,14] when determining porosity by the buoyancy method based on Archimedes’ principle. The porosity percentage and pore size are also affected by the position along the building direction, as shown in Figure 6. Therefore, further studies including the effect of this variable are necessary to determine the relative density of the parts more accurately.

The scanning speed was the most influential parameter, with a 74.86% contribution over porosity percentage. More specifically, the statistical model indicated a scanning speed of 700 mm/s as the best to achieve low levels of porosity. J. Liu et al. [9] found that at high scan speeds (1000 mm/s), the samples exhibited higher levels of porosity. For Shang et al. [10], scanning speeds that were too low and too high were both detrimental, resulting in increased porosity. However, in another study, laser power was found to have a greater influence on porosity than scanning speed [35]. In that work, laser energies from 200 to 280 W and scanning speeds between 400 and 600 mm/s were used, which differ from the ones chosen here for the production of the samples. It is well-known in the scientific community that both parameters are complementary, i.e., they act together to determine the energy density that the sample receives in its production [35]. The ranking of influence can vary and be affected by aspects such as the range in which each parameter is applied, the thickness of the layer chosen, the hatch spacing, etc. Therefore, the microstructural variations should be related and adjusted to the applied energy density that the sample receives and not to the value of each parameter individually.

The average roughness (Ra) of the top samples decreased with increasing energy densities. With higher energy densities, the molten tracks melted at a higher degree; this eliminated the valleys between them, thus obtaining a flatter and more uniform molten pool along the surface [11,36]. Woźniak et al. [20] also found the highest Ra value for samples produced with the lowest energy densities. 

The average hardness of the material increased from 190 to 259 HV, and the dispersion of results between successive indentations decreased with increasing energy density. The relationship between the hardness and the porosity of the sample was corroborated in the study of Cherry et al. [11] due to the variations in energy density. An average hardness of 235 HV was obtained in the work of Tolosa et al. [37]. In the work of Sistiaga et al. [38], a similar hardness value of 245 HV was measured for the as-built condition. The smaller grain sizes created by the high solidification rates contributed greatly to the increased hardness of the AM samples [9]. Those grains were characterized by a large fraction of low angle grain boundaries (LAGBs) [31]. The LAGBs acted as material strengtheners by blocking or hindering the movement of dislocations [9]. The samples studied in this project were characterized by having a high fraction of LAGBs that increased with increasing energy density. Therefore, using a higher energy density during the building process will produce parts with lower surface roughness and higher hardness; however, a balance must be found so as not to impair other microstructural properties. An overly high energy density produces samples with higher porosity and pore size, as has been demonstrated in this work.

**Figure 13:**
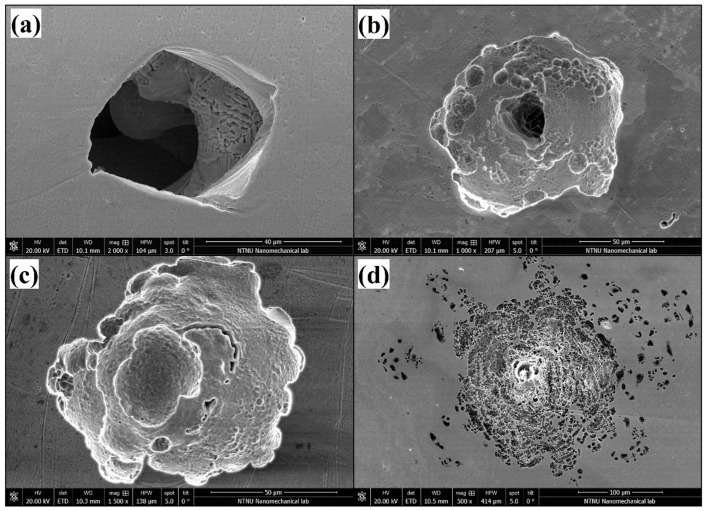
SEM images of a corroded pore of 52.17 J/mm^3^/2000× (**a**); pits around a pore of 89.44 J/mm^3^/1000× (**b**); shallow pit of 173.91 J/mm^3^/1500× (**c**); interconnected pits of 173.91 J/mm^3^/500× (**d**).

The group of samples produced with the lowest energy density exhibited poor corrosion resistance. This group was characterized by having E_pit_ values between 0.51 and 0.76 V and no ability to repassivate. The porosity values for this group were the highest, showing porosity percentages in the range of 7.8–12% and pore sizes between 31.1 and 37.9 μm. The high number of pits with unmelted powder particles were found in this group of samples. They were mainly located at the pores, starting from the inside and propagating the attack towards the surface, as shown in Figure 7a or Figure 13b. Suryawanshi et al. [19] stated that pores on the surface cause a preferential migration of chloride ions, thus acting as an ion deposit and accelerating corrosion. Sun et al. [18] explained that the accelerated metal dissolution was promoted by the diffusion barrier created on top of the pores, which led to a higher concentration of aggressive agents. In that work, values for breakdown potentials were found to decrease when increasing the percentage of porosity, which is also in line with what has been experienced in this work.

On the other hand, the energy density of 89.44 J/mm^3^ suggested by the powder supplier exhibited the highest corrosion resistance. The analyzed samples presented high values of E_pit_ between 1.08 and 1.14 V and the ability to recover the passive layer. This energy density resulted in fewer pores (<1%) with diameters of reduced size. Good corrosion resistance was also found in the samples produced with the highest energy density of 173.91 J/mm^3^. Samples from this group showed more shallow pits (Figure 13c). In contrast, wider corrosion attacks were found at this energy density, spreading the damage towards the vicinities via a network of interconnected porosities (Figure 13d).

Based on the polarization results, it can be seen that the AM materials show better corrosion resistance compared to the wrought samples. The reason for this behavior is that the pitting corrosion resistance is improved in AM samples in the pitting initiation step, as has already been shown by several authors [17,19,39]. 

In a new framework of the pitting corrosion proposed by Prof. Frankel and his co-workers [40], the pitting resistance of the materials could be under the control of the initiation or propagation step. In line with other researchers [17,19,39], their results show that the initiation step is more dominant here. The high cooling rates experienced by the AM samples are responsible for the annihilation or size reduction of the MnS inclusions, which are known to be related to the initiation of pitting [17,39]. The improved corrosion resistance identified in this work is expected to be due to a much lower concentration of sulfide inclusions and/or a reduced size of these inclusions in the AM specimens.

**Figure 14:**
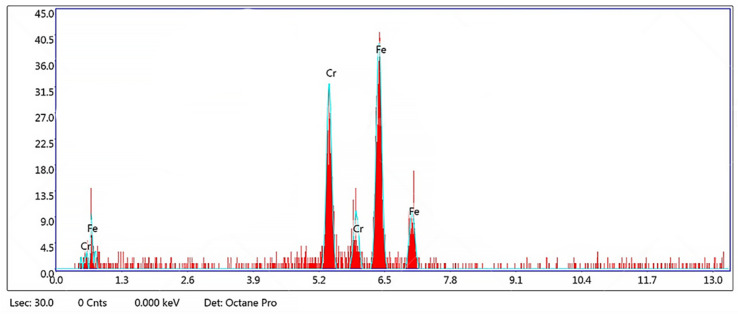
EDS spectrum of a pit of sample 5.2 after CPP measurement in 3.56 wt % NaCl at room temperature.

Chao et al. [39] concluded in their work that the higher corrosion resistance exhibited in the AM samples was due to a microstructure free of MnS inclusions. Similarly, in this work, the EDS analysis performed in some of those pits (Figure 14) did not reveal the presence of inclusions such as MnS, nor the existence of Si and Al oxides or Mo precipitations that were related to the reduction in corrosion resistance in previous works [41,42].

## 5. Conclusions

Corrosion and microstructural investigations were carried out via the Selective Laser Melting (SLM) process on 316L stainless steel produced by additive manufacturing. This work generated the following conclusions.

- The position along the building in the Z-direction influences the amount of porosity and pore size on a printed component, both increasing for higher positions on the *Z* axis.
- The highest porosity was found in the samples produced with low energy densities (52.17 J/mm^3^). When increasing that value to 89.44 J/mm^3^, the porosity decreased. However, it increased again for higher energy densities (173.91 J/mm^3^). The Taguchi model suggested 99.38 J/mm^3^ as the optimal energy density to produce samples with low levels of porosity (0.05%).
- The ANOVA confirmed the scanning speed as the most influential factor in reducing the porosity percentage (74.9% reduction), followed by the position along the *Z* axis (22.8%), and finally, the laser energy (2.3%).
- The average surface roughness (Ra) of the AM samples decreased linearly from 11 to 4.6 µm, with increasing energy densities. The hardness (HV) increased drastically between the lowest energy densities, and stabilized around ~253 HV for energy densities >74.53 J/mm^3^. The area fraction of large grains increased with increasing energy densities as well as the low-angle grain boundaries.
- The cyclic potentiodynamic polarization curves confirmed the higher corrosion resistance of the AM samples compared to wrought samples. More positive breakdown potentials and repassivation potentials were found in the AM samples with porosity levels <1%. The pores were found to be preferred pit initiation sites, as revealed by the SEM images. In samples with high porosity, most pits originated within the pores, spreading the corrosion attack towards the vicinity via a network of interconnected porosities. No inclusions, such as MnS or other elements that could be detrimental to the corrosion resistance of the AM samples, were found in the EDS analysis.

The morphology and number of pits in the samples were observed to have varied according to the energy density used for their production. This has been confirmed visually through the images obtained in the SEM; however, carrying out a more detailed study would be desirable by weighting the samples or measuring the profile of the pits, thus obtaining specific values of depth and width for each applied energy density. Moreover, the literature has also shown that precipitates may appear at the grain boundaries after solution heat treatment [43]. Studying the pros and cons of post-heat treatment on the microstructure and corrosion resistance would be interesting for a further study. Some of the limitations were the number of process parameter combinations studied; it would be convenient to extend and process these with a higher performance statistical software in order to obtain a greater amount of significant data.

## Figures and Tables

**Table 1:** Chemical composition of 316L (powder) according to DIN EN10088-3.

Element	C	Cr	Ni	Mo	Si	Mn	S	Fe
**Mass fraction (%)**	0.03	16.5–18	10–13	2–2.5	0–1	0–2	0–0.03	Balance

**Table 2:** Combination of the different processing parameters with their values and the resulting energy density (J/mm^3^).

Laser Energy (W)	Hatch Spacing (mm)	Layer Thickness (mm)	Scanning Speed (mm/s)	Energy Density (J/mm^3^)	No. Runs
150	0.115	0.025	400	130.43	1
			700	74.53	2
			1000	52.17	3
180	0.115	0.025	400	156.52	4
			700	89.44	5
			1000	62.61	6
200	0.115	0.025	400	173.91	7
			700	99.38	8
			1000	69.56	9

**Table 3:** Input factors L9 (OA) and their levels.

Factors	Units	Levels		
		1	2	3
Laser energy	W	150	180	200
Scanning speed	mm/s	400	700	1000
Position (XOZ)	#	2	5	8

**Table 4:** Characteristic corrosion values and porosity (%) for the examined samples subjected to the CPP test.

Sample	E_corr_	E_pit_	E_rep_	i_pass_	Porosity (%)
3.2	−0.1054	0.7637	−0.0634	1.63 × 10^−6^	12.01
3.5	−0.0694	0.5154	−0.0339	3.91 × 10^−6^	8.16
3.8	−0.1127	0.7050	−0.1107	4.18 × 10^−6^	7.74
5.2	0.0085	1.0807	*	0.92 × 10^−6^	0.72
5.5	−0.0566	1.1325	0.5410	1.31 × 10^−6^	0.43
5.8	−0.0772	1.1440	*	0.86 × 10^−6^	0.26
7.2	−0.1193	0.9814	0.5573	1.00 × 10^−6^	1.10
7.5	−0.0682	1.1187	0.3968	0.75 × 10^−6^	0.84
7.8	−0.0825	1.1447	0.1573	1.04 × 10^−6^	0.47
CM	0.0085	0.3831	0.1690	4.71 × 10^−6^	0.00

Current (log i): (A/cm^2^); Potential (E): (V vs. Ag/AgCl); *: no value obtained.

**Table 5:** Taguchi array 9 × 9, experimental results and their S/N ratios.

Exp. Runs	Factors			Experimental Results	S/N Ratios of Results
	$\;\;\;\;P_{eff}$	$\;\;\;v_{s}$	# (XOZ)	Porosity (%)	Porosity (dB)
1	150	400	2	0.27	11.3727
2	150	700	5	0.07	23.098
3	150	1000	8	7.74	−17.7748
4	180	400	5	0.09	20.9151
5	180	700	8	0.26	11.7005
6	180	1000	2	1.57	−3.918
7	200	400	8	0.47	6.558
8	200	700	2	0.08	21.9382
9	200	1000	5	0.92	0.7242

**Table 6:** Mean S/N ratio response table for porosity (%).

Symbol	Process Parameters	Mean S/N Ratio				
		Level 1	Level 2	Level 3	Max–Min	Rank
$P_{eff}$	Laser energy (W)	5.565	9.565	9.74	4.175	3
$v_{s}$	Scanning speed(mm/s)	12.948	18.912	−6.989	25.901	1
#	Position (XOZ)	9.797	14.912	0.161	14.751	2

**Table 7:** Confirmation test results for porosity (%).

	Optimal Process Parameters	Optimal Process Parameters
	(Supplier)	(Taguchi)
Level	$P_{eff\left(180\right)}-v_{s\left(700\right)}-{\#}_{\left(5\right)}$	$\;P_{eff\left(200\right)}-v_{s\left(700\right)}-{\#}_{\left(5\right)}$
Porosity (%)	0.43	0.05
Percentage reduction of porosity	88.37%	

**Table 8:** Confirmation results for the fitted model.

Factors	Degree of Freedom	Sum of Squares	Mean Squares	%Contribution
Laser energy Scanning speed Position Total	2 2 2 8	33.46 1104.00 336.62 1474.73	16.732 551.999 168.310	2.268% 74.861% 22.825%

**Table 9:** ANOVA of porosity (%) showing the residuals for the adjusted model.

Run	Experimental	Predicted	Residuals
	Porosity (%)	Porosity (%)	Porosity (%)
1	7.75	7.62	0.13
4	0.08	0.01	0.07
7	0.34	0.13	0.21
9	0.45	0.43	0.02

## Data Availability

Not applicable.
